# Phosphatidylinositol 3-Kinase/Protein Kinase B/Mammalian Target of the Rapamycin Pathway-Related Protein Expression in Lung Squamous Cell Carcinoma and Its Correlation with Lymph Node Metastasis

**DOI:** 10.1155/2022/4537256

**Published:** 2022-08-23

**Authors:** Fang Shi, Ling Li

**Affiliations:** ^1^Department of Oncology, Jiangxi Chest Hospital, Nanchang 330006, Jiangxi, China; ^2^Department of Oncology, Second Affiliated Hospital of Nanchang University, Nanchang 330006, Jiangxi, China

## Abstract

The targeted therapy of lung squamous cell carcinoma (LSCC), a pathological type of non-small-cell lung cancer, is relatively lacking by contrast with lung adenocarcinoma. The overexpression or inhibition of intracellular signaling pathways leads to disease. To evaluate genes for a targeted therapy in LSCC, we analyzed PI3K pathway components in LSCC tissues and found elevated PI3K levels in LSCC tissues compared with adjacent counterparts. A comparison of PI3K levels in tissues with and without metastasis revealed that the PI3K pathway activity was greatly increased in metastatic tissues. Our findings suggest that the metastasis of cancer cells in patients with LSCC is closely related to the overexpression of PI3K pathway components in cancer tissues. We performed *in vitro* cell culture experiments and found that inhibition of PI3K activity decreased proliferation and increased apoptosis in H520 cells, suggesting that PI3K pathway inhibition limits LSCC cell proliferation. We hypothesize that LSCC metastasis is related to the overexpression of PI3K pathway components and inhibiting this pathway may help reduce the risk of lymph node metastasis in LSCC patients.

## 1. Introduction

Lung cancer, which bears responsibility for 18% of cancer deaths worldwide, is a malignancy ranking first in morbidity and mortality [1, 2]. Non-small-cell lung cancer, as the most common type, accounts for about four-fifths of the total number of lung cancers; lung squamous cell carcinoma (LSCC) and lung adenocarcinoma, they are two common pathological types of non-small-cell lung cancer [3, 4]. With the rapid development of gene detection and targeted therapy technologies, the early diagnosis rate and treatment effects of lung adenocarcinoma have greatly improved. Because most LSCCs have no clear driver genes, the progress of a targeted therapy is relatively lagging behind that of lung adenocarcinoma. Traditional radiotherapy and chemotherapy are mostly performed to treat patients with LSCC [5, 6]. However, LSCC is dominated by central type lung cancer, which involves large blood vessels. Moreover, the cancer tissue is volatile and necrotic and forms cancerous cysts. In addition, the patients are mostly elderly and most are staged late at the time of diagnosis, resulting in limited benefits of traditional radiotherapy and chemotherapy [7, 8]. Some patients with LSCC have a shorter progression-free survival period after traditional treatment. This makes the exploration of targeted therapy of genes and related sites in LSCC a hot spot for lung cancer prevention and treatment research.

The occurrence of cancer is related to the abnormal proliferation of cells, and the signaling pathways can affect both cell proliferation and apoptosis by transmitting signals inside and outside the cell [9, 10]. Zhou et al. confirmed that inhibiting the activity of the Stat3 pathway can reduce the activity of the microglia, thereby reducing the inflammatory response in patients with *Mycobacterium* infection [11]. Hong et al. reported that disturbances in meiosis-related pathways can reduce male sperm concentration [12]. Intracellular pathways are closely related to multiple processes of cell growth and metabolism. PI3K/Akt/mTOR, an important pathway for regulating cell growth in cells, affects the proliferation of cancer cells in breast, ovarian, and bladder cancer and other malignant tumors [13–15]. Pathway components include phosphatidylinositol 3-kinase (PI3K), an upstream protein, that has both phosphatidylinositol kinase and serine/threonine activities and can be activated by various nerve and growth factors [16]. When PI3K is activated, it accumulates in the phosphorylated plasma membrane of the receptor and catalyzes the conversion of phosphatidylinositol diphosphate on the surface of the plasma membrane to triphosphate, which can act as a messenger to activate the expression of downstream proteins [17]. Protein kinase B (Akt), a downstream protein in the PI3K/Akt/mammalian target of the rapamycin (mTOR) axis, has three subtypes, namely, Akt 1, 2, and 3, which have different regulatory effects on cells [18, 19]. mTOR is a regulatory molecule downstream of the PI3K/Akt/mTOR axis and cannot only maintain the steady state of pathway conduction but also controls the pathway's energy metabolism and immune regulation [20, 21]. Scholars such as Xia et al. [22] believe that the PI3K/Akt/mTOR axis is essential in breast cancer metastasis. Chen et al. [23] and other scholars found in a study to find biomarkers of early cervical cancer with lymph node metastasis (LNM) that PI3K/Akt/mTOR significantly affected LNM in patients with early cervical cancer. In addition, studies have shown that PI3K/Akt/mTOR axis activation promotes human pancreatic neuroendocrine tumor growth and development [24]. It can be seen that this axis is strongly linked to the occurrence and metastasis of various malignant tumors. If the association between this pathway and the occurrence and metastasis of LSCC can be found, it will provide new ideas for LSCC prevention and treatment. However, its expression and mechanism of action in LSCC cells remain unexplored. Therefore, PI3K pathway levels in LSCC tissues and their correlation with LNM were investigated to provide insights into the treatment of LSCC.

## 2. Data and Methods

### 2.1. LSCC Tissue Specimens, Cell Lines, and Main Reagents

We collected cancerous tissues and corresponding counterparts (<3 cm away from cancerous tissues) as well as normal lung epithelial tissues (>5 cm away from cancerous tissues) from 10 LSCC patients. In addition, the cancer tissues of 3 LSCC patients with LNM and 3 LSCC cases without LNM who were treated in our hospital during the same period were collected. The LSCC patients of this study are from the Jiangxi Chest Hospital and the Second Affiliated Hospital of Nanchang University. The patients were diagnosed with LSCC using histopathology, and they were undergoing surgery for the first time. Radiotherapy and chemotherapy were not performed before surgery. We obtained the patient's consent before performing the experiments and collecting tissues.

Human LSCC cell line H520 was provided by Shanghai Guandao Biotechnology Co., Ltd. (Item No.: GD-C2318717), and human normal lung epithelial cell BEAS-2B was provided by Shanghai Yiyan Biotechnology Co., Ltd. (Item No. EY-X1008). The PI3K pathway inhibitor LY294002 was provided by Beijing Biolab Technology Co., Ltd. (Item No.: M00003). Trizol reagent was provided by Shenzhen Zike Biotechnology Co., Ltd. (Item No.: 15596018). The (Cell Counting Kit-8 (CCK-8) kit was provided by Beijing Soleibo Technology Co., Ltd. (Item No.: CA1210-500T). RIPA lysate was provided by Shanghai Biyuntian Company (Item No.: P0013 K). Diethyl pyrocarbonate (DEPC) was provided by Sigma Company of the United States (Item No.: V900882). The constant temperature incubator was provided by Beijing Yongguang Medical Instrument Co., Ltd. (Item No.: DHP-500). The low-speed centrifuge was provided by Shanghai Fuller Biotechnology Co., Ltd. (Item No.: SG-1063-FL). The real-time fluorescence quantitative polymerase chain reaction (PCR) instrument was provided by Applied Biosystems, USA (Item No.: V115896).

### 2.2. CCK-8 Method

The CCK-8 kit was adopted for cell proliferation determination, and the fine suspension was seeded in a 96-well plate, according to the kit's instructions. Cell density was maintained at 2,000 cells/well. The inoculated cells were then placed in a constant temperature (37°C) incubator for 4 h of culture. Then, grouping treatments were performed. The H520 group did not receive any treatment. To the H520 + LY294002 group, 10 nM LY294002 was added at a concentration of 10 *μ*mol/L. The Petri dishes were placed in an incubator and maintained at a constant temperature for 24 h. CCK-8 was slowly added to the Petri dishes, which were then sent to the incubator for 2 h of incubation. Finally, a microplate reader was used to detect cell absorbance value (A450), and cell proliferation was evaluated according to the absorbance.

### 2.3. Flow Cytometry (FC) Detection

We placed the cells in a 96-well plate and set 5 multiple wells for each cell (100 *μ*L per well). Cell density was maintained at 2,000 cells/well. The grouping process was the same as that for the CCK-8 method. We then added 5 *μ*L of Annexin-FITC, as well as PI staining solution of the same volume to the processed cell suspension and gently shook the cells until the staining solution and cell suspension were thoroughly mixed. The mixed cell suspension was incubated for 30 min under dark conditions, and cell apoptosis was detected by FC within 30 min after incubation.

### 2.4. Western Blot (WB) Detection

The level of protein was detected by Western blot. The required materials include HOXA10 rabbit anti-rat primary antibody, general-purpose WB antibody diluent (volume ratio of 1 : 1000), and rabbit anti-rat IgG-labeled secondary antibody (Beijing Boaosen Biotechnology Co., Ltd., the product number is bs-2502R, C05–07001, and C05-07002, respectively). The specific steps are centrifuge the cells cultured in the radioimmunoprecipitation assay (RIPA) lysate (10,000 × g, 10 min), and take a portion of the supernatant in order to detect the protein concentration using a protein kit. Another portion of the supernatant was boiled (100°C) for 5 min and electrophoresis was performed after protein denaturation. We loaded 20 *μ*g protein per lane and performed electrophoresis. Then, the separated proteins were transferred to the mold and placed in 5% milk powder soaked for 1 h and then stored at 4°C. After 12 h, the membrane was treated with TBST washing, secondary antibody incubation (1 h), and TBST rinsing twice. The ECL chemiluminescence reagent was added to the membrane and allowed in the dark. The gel imaging system was used to expose and take pictures and to analyze the gray value of the protein band.

### 2.5. Nano-qPCR Detection of mRNA Expression

To the cells placed in an RNA-free EP tube, 1 mL of the Trizol reagent was added. The cells were gently pipetted to mix the two thoroughly and the Trizol method was used to extract the total RNA. First, a spectrophotometer was used to detect RNA concentration and purity, and following this, reverse transcription of total RNA into cDNA was performed. We referred to the PCR kit's instructions to configure the reaction system. The reaction system in this study included 10 *μ*L 2 × SYBR Premix Taq II, 0.4 *μ*L sense and antisense primers, and a 2 *μ*L cDNA template. DEPC water was added to 20 *μ*L, and finally, 5 *μ*L of gold nanoparticles (concentration 0.5 *μ*M) were added. The 2^−△△Ct^ formula was applied to calculate relative gene expression relative to GAPDH with the average Ct value obtained.

### 2.6. Statistical Analysis

GraphPad Prism 8.3 and SPSS 23.0 were employed for data analysis. Normally, distributed quantitative data were denoted by mean ± standard deviation. Multigroup comparisons and pairwise comparisons between groups were performed using one-factor analysis of variance and the SNK-Q test, respectively, with *P* < 0.05 as the significance level.

## 3. Results and Discussion

### 3.1. PI3K Pathway Levels in Different Tissues

**Figure 1:**
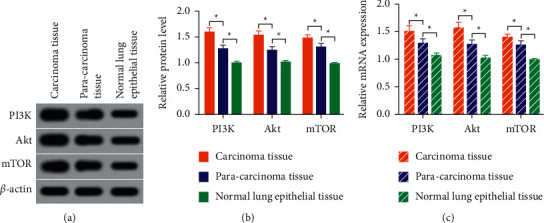
The levels of phosphatidylinositol 3-kinase (PI3K) pathway proteins in different tissues. (a) Gray values of phosphatidylinositol 3-kinase/protein kinase B (Akt)/mammalian target of rapamycin (mTOR). (b) Relative protein levels of PI3K pathway. (c) Relative mRNA levels of PI3K pathway. ^∗∗^*P* < 0.05.

By comparing the levels of PI3K pathway proteins in cancer tissues and adjacent counterparts, as well as normal lung epithelial tissues, we found that PI3K, Akt, and mTOR protein levels were higher in adjacent tissues when compared to normal lung epithelial cells (*P* < 0.05) and were much higher in cancer tissues compared with adjacent counterparts (*P* < 0.05), as shown in Figures 1(a) and 1(b). In addition, the relative mRNA expressions of PI3K, Akt, and mTOR proteins were higher in paracancerous tissues versus normal lung epithelial cells (*P* < 0.05). Besides, higher PI3K, Akt, and mTOR mRNA levels were determined in cancer tissues compared with adjacent ones (*P* < 0.05, Figure 1(c)).

### 3.2. PI3K Pathway-Related Proteins in Metastatic and Nonmetastatic Tissues

**Figure 2:**
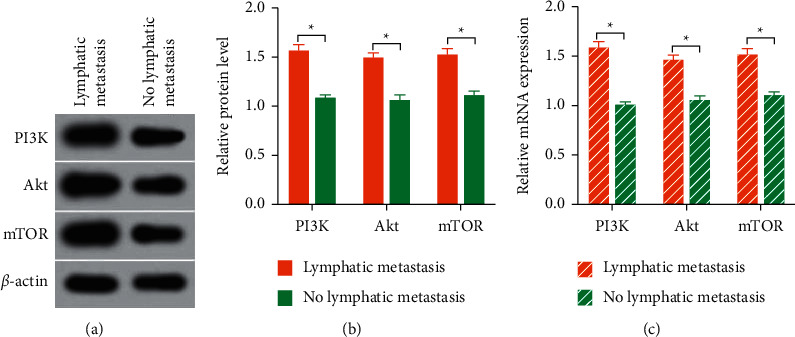
Phosphatidylinositol 3-kinase (PI3K) pathway-related proteins in tissues with and without metastasis. (a) The gray value of proteins related to the PI3K pathway. (b) The relative proteins levels of the PI3K pathway. (c) Relative mRNA levels of PI3K pathway. Akt: protein kinase B; mTOR: mammalian target of rapamycin. ^*∗*^*P* < 0.05.

The relative protein expressions of PI3K, Akt, and mTOR were high in metastatic tissue and level low in nonmetastatic tissues, as shown in Figures 2(a) and 2(b). In addition, the relative mRNA expressions of PI3K, Akt, and mTOR were high in metastatic tissue and level low in nonmetastatic tissue as shown in Figure 2(c).

### 3.3. Levels of PI3K in Different Cells

**Figure 3:**
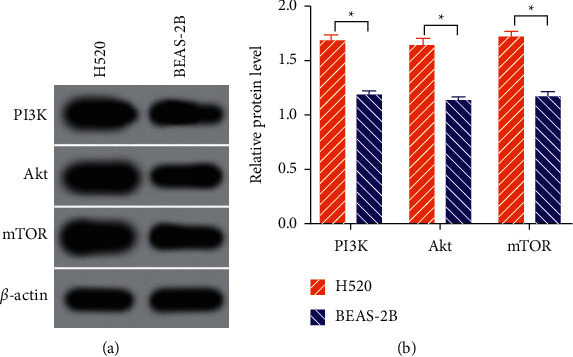
Phosphatidylinositol 3-kinase (PI3K) pathway proteins in H520 and BEAS-2B cells. (a) Gray values of PI3K pathway proteins. (b) Relative levels of PI3K pathway proteins. Akt: protein kinase B; mTOR: mammalian target of rapamycin. ^*∗*^*P* < 0.05.

*In vitro* cell experiments were carried out to investigate the mechanism underlying PI3K pathway activation in LSCC initiation and progression. Relative expressions of PI3K, Akt, and mTOR were found to be much higher in H520 cells than in BEAS-2B (*P* < 0.05) as shown in Figures 3(a) and 3(b).

### 3.4. Impact of PI3K Inhibitor on Cells

**Figure 4:**
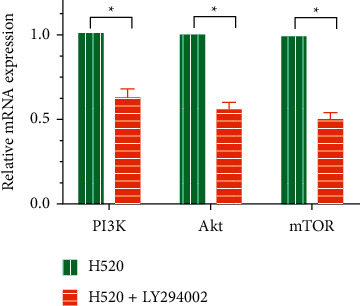
Relative PI3K, Akt, and mTOR mRNA levels in H520 cells. ^*∗*^*P* < 0.05.

**Figure 5:**
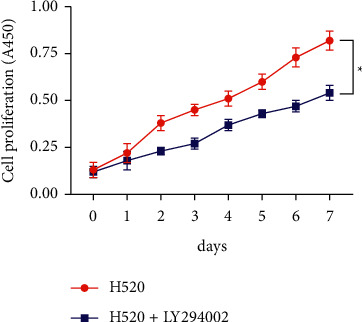
Impact of LY294002 (a PI3K inhibitor) on cell proliferation. ^*∗*^*P* < 0.05.

**Figure 6:**
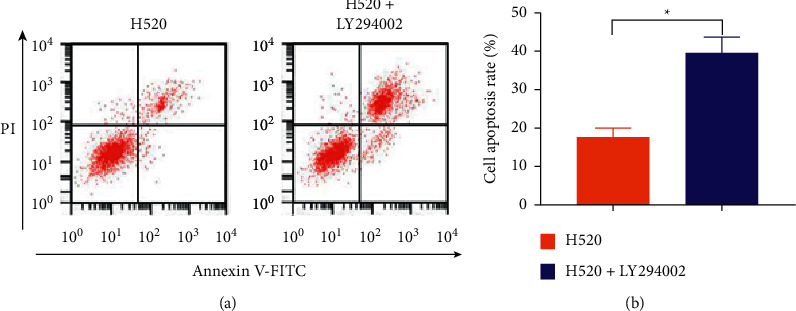
Impact of inhibitors on apoptosis. (a) Apoptosis histogram of flow cytometry. (b) Comparison of the apoptosis rate. ^*∗*^*P* < 0.05.

LY294002, a PI3K inhibitor, greatly reduced PI3K, Akt, and mTOR mRNA levels in H520 cells, as shown in Figure 4, suggesting that PI3K pathway activity decreased after LY294002 treatment. Results from the CCK-8 assay to detect cell viability and proliferation revealed that inhibition of the PI3K pathway reduced the viability and proliferation ability of H520 cells (Figure 5). In addition, we found that the apoptosis rate of H520 cells increased after the activity of the PI3K pathway decreased as shown in Figures 6(a) and 6(b).

## 4. Discussion

Understanding the mechanism of metastasis in patients with LSCC can help reduce the risk of death. Tissue homeostasis has been shown to be important for physical health [25, 26]. In a healthy human body, the body environment remains relatively stable under the coordinated operation of the nervous system, circulatory system, immune system, and various organs. When the body is stimulated by external damage or internal therapeutic factors, multiple signaling pathways are activated or inhibited due to the stress response, lowering the relative stability of the tissue and leading to the occurrence of diseases [27–29].

In the PI3K/Akt/mTOR axis, PI3K, as an upstream protein, can be categorized into three types, namely, I, II, and III, according to the structure. All three types have a regulatory effect on cancer cell growth and movement [30]. Type I PI3K is the most important. Curigliano reported that type I PI3K is hyperactivated in patients with solid malignancies and hematological malignancies [31]. The PI3K/Akt/mTOR axis is essential in abnormally proliferated ovarian cancer cells, and using pathway inhibitors to reduce pathway activity is expected to improve therapeutic effects in patients with ovarian cancer [14]. Costa et al. reported that PI3K/Akt/mTOR can be used as a new area of targeted therapy and drug development for triple-negative breast cancer [13]. Therefore, exploring the correlation between the levels of the PI3K pathway in LSCC and its metastasis to the lymph node may provide new insights for the targeted therapy of LSCC.

We compared the expression of PI3K pathway proteins in different tissues (including cancer tissues, adjacent tissues, normal tissues, and lymph node metastatic tissues and nonmetastatic tissues) and found statistically higher PI3K, Akt, and mTOR protein levels in cancer tissues compared with adjacent and normal tissues. Moreover, statistically higher PI3K, Akt, and mTOR protein levels were determined in metastatic tissues compared with nonmetastatic tissues. Thus, LNM of LSCC may be associated with the PI3K, Akt, and mTOR overexpression in the cells. Furthermore, the mechanism of the PI3K pathway in LSCC initiation and progression was investigated *in vitro*. PI3K, Akt, and mTOR protein levels in LSCC cells (H520) were observed to be statistically elevated compared with normal lung epithelial BEAS-2B cells. While PI3K, Akt, and mTOR mRNA expression in H520 cells treated with the PI3K inhibitor LY294002 was greatly decreased. Thus, PI3K pathway activity decreased after LY294002 treatment. We used the CCK-8 assay to detect cell activity and proliferation and found decreased H520 cell activity and proliferation ability after the PI3K pathway activity decreased. Moreover, the rate of apoptosis in H520 cells increased after the PI3K pathway activity decreased. Therefore, we believe that the metastasis of LSCC cells may be related to the overexpression of PI3K pathway components.

## 5. Conclusion

In summary, we compared the levels of PI3K axis components in LSCC tissues and normal lung epithelial tissues and found statistically higher levels in LSCC tissues compared with normal tissues. Comparing the levels of PI3K axis components in tissues with and without metastasis, we found that the PI3K pathway activity was greatly increased in metastatic tissues, suggesting that the overexpression of PI3K pathway proteins is closely related to metastasis in patients with LSCC. Through *in vitro* cell experiments, we found that inhibiting the PI3K pathway activity led to decreased cell proliferation ability and increased apoptosis rate. We believe that the metastasis of LSCC may be linked to the overexpression of the PI3K pathway, and inhibiting the activity of the pathway may help reduce the risk of metastasis in patients with LSCC.

## 6. Strengths and Limitations

In this study, it was found through experiments that the LNM of LSCC cells is linked to the overexpression of PI3K axis-related proteins in cancer tissues, and inhibiting the PI3K/Akt/mTOR axis activity may help reduce the risk of LNM in LSCC patients. This provides new ideas and a theoretical basis for the prevention and treatment of LSCC. However, this study also has some shortcomings, such as the limited indicators detected, the phosphorylation levels of PI3K, Akt, and mTOR-related proteins could not be detected, and the depth of the related mechanisms needs to be deepened. In the future, various indicators need to be detected and the mechanism of action of the PI3K/Akt/mTOR axis needs to be further studied and discussed, which will help to further understand the role played by this axis in the occurrence, development, and treatment of the disease and provide a more comprehensive theoretical support for the prevention and treatment of LSCC.

## Data Availability

The labeled dataset used to support the findings of this study is available from the corresponding author upon request.
